# Role of Downregulation and Phosphorylation of Cofilin in Polarized Growth, MpkA Activation and Stress Response of *Aspergillus fumigatus*

**DOI:** 10.3389/fmicb.2018.02667

**Published:** 2018-11-02

**Authors:** Xiaodong Jia, Xi Zhang, Yingsong Hu, Mandong Hu, Xuelin Han, Yansong Sun, Li Han

**Affiliations:** ^1^Institute for Disease Control and Prevention of PLA, Beijing, China; ^2^Comprehensive Liver Cancer Center, Beijing 302 Hospital of PLA, Beijing, China

**Keywords:** *Aspergillus fumigatus*, cofilin downregulation, cofilin phosphorylation, polarized growth, cell wall integrity, stress response

## Abstract

*Aspergillus fumigatus* causes most of aspergillosis in clinic and comprehensive function analysis of its key protein would promote anti-aspergillosis. In a previous study, we speculated actin depolymerizing factor cofilin might be essential for *A. fumigatus* viability and found its overexpression upregulated oxidative response and cell wall polysaccharide synthesis of this pathogen. Here, we constructed a conditional *cofilin* mutant to determine the essential role of cofilin. And the role of cofilin downregulation and phosphorylation in *A. fumigatus* was further analyzed. Cofilin was required for the polarized growth and heat sensitivity of *A. fumigatus*. Downregulation of cofilin caused hyphal cytoplasmic leakage, increased the sensitivity of *A. fumigatus* to sodium dodecyl sulfonate but not to calcofluor white and Congo Red and farnesol, and enhanced the basal phosphorylation level of MpkA, suggesting that cofilin affected the cell wall integrity (CWI) signaling. Downregulation of cofilin also increased the sensitivity of *A. fumigatus* to alkaline pH and H_2_O_2_. Repressing cofilin expression in *A. fumigatus* lead to attenuated virulence, which manifested as lower adherence and internalization rates, weaker host inflammatory response and shorter survival rate in a *Galleria mellonella* model. Expression of non-phosphorylated cofilin with a mutation of S5A had little impacts on *A. fumigatus*, whereas expression of a mimic-phosphorylated cofilin with a mutation of S5E resulted in inhibited growth, increased phospho-MpkA level, and decreased pathogenicity. In conclusion, cofilin is crucial to modulating the polarized growth, stress response, CWI and virulence of *A. fumigatus*.

## Introduction

*Aspergillus fumigatus* is an important pathogenic fungus and causes 90% of aspergillosis. Themortality rate of invasive aspergillosis (IA), the severest aspergillosis, is up to 90% (Dagenais and Keller, 2009). The virulence of *A. fumigatus* refers to multi-factors (Li et al., 2016; Shemesh et al., 2017). It has been reported that the actin-cytoskeleton regulatory proteins are involved in virulence of *A. fumigatus* and other fungi (Renshaw et al., 2016). Besides, the actin-cytoskeleton regulatory proteins of fungi also play a role in spore production, hyphal growth, stress response, cell wall integrity (CWI). In *Candida albicans*, deletion of actin-related protein Sac1 results in defect of hyphal growth and biofilm, increased sensitivity to cell wall stressors and hypovirulence (Zhang et al., 2015); deletion of actin-related protein Arp2 abolishes hyphal development to form round and swollen yeast cells and becomes hypovirulent (Epp et al., 2010). In *Cryptococcus neoformans*, Wsp1 protein promotes actin assembly and its mutation results in defects in growth, chitin distribution, endocytosis, exocytosis, and hypovirulence (Shen et al., 2011). In *Botrytis cinerea*, deletion of F-actin capping protein BcCPA1 severely influences hyphal growth and morphology, and virulence (Gonzalez-Rodriguez et al., 2016). In *Magnaporthe oryzae*, deletion of the actin-regulating kinase homolog MoArk1 (Δ*MoArk1*) displays hyphal growth defect and affects CWI. Δ*MoArk1* has increased resistance to oxidative stress and decreased virulence on rice and barley (Wang et al., 2013). In *Fusarium graminearum*, deletion of actin-bundling protein FgFim (Δ*FgFim*) reduces the growth rate and forms irregular hyphae. Besides, Δ*FgFim* attenuates virulence and exhibits increased sensitivity to cell wall and oxidative stress (Zheng et al., 2014). To the best of our knowledge, few studies on actin-cytoskeleton regulatory protein in *A. fumigatus* have been reported. Renshaw et al. (2016) have recently showed that deletion of myosin B and myosin E of *A. fumigatus* displays abnormal septation, reduced growth, increased sensitivity to cell wall stressors and hypovirulence.

As an actin-binding protein, cofilin belongs to actin depolymerizing factor (ADF)/cofilin family (15–20 kDa) and plays a conserved role in actin cytoskeleton dynamic (Moon and Drubin, 1995). Only one isoform of cofilin is expressed in yeast. Deletion of cofilin is lethal for yeast. The function of cofilin in yeast is studied by constructing site-directed mutants (Lappalainen et al., 1997). The yeast cofilin is involved in endocytosis, sorting of the soluble secretory proteins, environmental challenge and multi-drug resistance (Chen and Pollard, 2011; Curwin et al., 2012; Kotiadis et al., 2012; Henriques et al., 2015). However, the effect of downregulation of cofilin in yeast is unknown. In mammalian cells, cofilin has two isoforms (cofilin-1 and cofilin-2) and is involved in various physiological functions including cell locomotion (Ghosh et al., 2004; Bravo-Cordero et al., 2013), mitochondrial-mediated apoptosis (Chua et al., 2003; Klamt et al., 2009) cellular stress responses (Thirone et al., 2009) and pathological situations (Bamburg and Wiggan, 2002). The depolymerizing activity of cofilin is mainly regulated by the serine phosphorylation, alkaline pH, phosphoinositides and other actin-binding proteins (Moon and Drubin, 1995; Lappalainen et al., 1997; Bernstein and Bamburg, 2010; Bao et al., 2015). However, the activity of yeast cofilin couldn’t be regulated by pH (Bernstein and Bamburg, 2010).

Recently, we have constructed a *cofilin* overexpressing strain (*cofilin OE*) and found that overexpression of cofilin in *A. fumigatus* could increase the resistance to oxidative stress, and change the cell wall components and host inflammatory response. However, cofilin overexpression didn’t influence polarized growth of *A. fumigatus*. We failed to delete the *cofilin* gene of *A. fumigatus* with several strategies and no strain was survival, which hinted that loss of cofilin may lead to the death of *A. fumigatus* (Jia et al., 2017).

To further explore the function of cofilin in *A. fumigatus*, we first established a strain conditionally expressing the *cofilin* under the control of doxycycline-controlled *tet-on* promoter in this study. Our study using this strain showed that cofilin was essential for viability of *A. fumigatus*. Downregulation of cofilin in *A. fumigatus* resulted in impaired polarized growth and CWI, increased sensitivity to alkaline pH and oxidative stresses, and hypovirulence. Intriguingly, cofilin phosphorylation also plays a critical role on the growth and MpkA activation of *A. fumigatus*.

## Materials and Methods

### Strains, Culture Conditions, and Chemicals

The *A. fumigatus* strains used in this work are listed in Supplementary Table S1. The non-homologous end-joining deficient *A. fumigatus* strain CEA17*Δku80* (da Silva Ferreira et al., 2006) served as wild-type strain in this study for all *in vitro* and animal model experiments. Calcofluor white 28 (F3543-1G), Lysing Enzymes from *Trichoderma harzianum* (L1412-5G) and *trans, trans*-farnesol (277541-1G) were purchased from Sigma–Aldrich. The monoclonal antibodies of MpkA (9102), p-MpkA (4370) were purchased from Cell Signaling Technology. The anti-Histone H3 monoclonal antibody (HX1850) was purchased from Huaxingbio in China. The polyclonal antibody of cofilin was purchased from ABZYMO Biosciences in China.

### Phylogenetic Analysis (Winkelstroter et al., 2015)

The sequence of *A. fumigatus* cofilin protein was obtained from the PubMed protein database^1^. And the cofilin sequences of other species were obtained by alignment to *A. fumigatus* cofilin using PubMed Blastp. As the cofilin functions of yeast, *Mus musculus* and *Homo sapiens* have been reported, we selected their cofilin sequences for alignment. The phylogenetic analysis was performed by using MEGA 5.0 software. The alignment was performed with ClustalW and manually curated. The evolutionary history was inferred using the Neighbor-Joining method.

### Construction of the Conditional *Cofilin* Mutant Strain (*Cofilin_teton_*)

The name and sequence of *cofilin* (AFUA_5G10570) gene were determined from the PubMed gene database. The *cofilin_teton_* was generated using a modified method based on homologous recombination as described previously (Dichtl et al., 2012). First, the pyrithiamine resistance cassette and the tet-on system were amplified with the primer pair coftj-tetonS and coftj-tetonA using pCH008 (Helmschrott et al., 2013) as template. The upstream fragment (position -1090 ∼-22) of *cofilin* gene and 1426 bp downstream fragment beginning with the start codon were amplified using the genome DNA (gDNA) of non-homologous end-joining deficient strain CEA17*Δku80* as template with the primer pairs coftj-upS and coftj-upA, coftj-dwS and coftj-dwA, respectively. Then the conditional *cofilin* mutant cassette was constructed by fusion PCR and purified for transformation. The protoplasts of CEA17*Δku80* strain were generated by Lysing Enzymes (L1412, Sigma). The cassette was transformed into protoplasts in the presence of polyethylene glycol (PEG). The transformants were screened on Aspergillus minimal medium (AMM) plates containing 1.2 M sorbitol, 100 μg ml^-1^ doxycycline and 0.1 μg ml^-1^ pyrithiamine.

### Construction of the *Cofilin^S5A^* Mutant Strain

A parental cassette without mutation of cofilin was firstly constructed as shown in Supplementary Figure S3. The cassette included upstream region, middle region and downstream region. The upstream region including 5′ flanking sequence (-1215 bp ∼-1), *cofilin* ORF (ATG∼TAG) was amplified from gDNA with primers cofsite-m-upS and cofsite-m-upA. The middle region including only the ptrA sequence was amplified from pJW103 with the primers cofsite-m-ptrAs and cofsite-m-ptrAa. The downstream including 3′ flanking sequence of *cofilin* gene was amplified from gDNA with primers cofsite-m-dwS and cofsite-m-dwA. Three regions were fused to construct the parental cassette with primers cofsite-m-upS and cofsite-m-dwA. Then a *cofilin^S5A^* mutant cassette was constructed by inserting S5A mutation of *cofilin* in the parental cassette. The *cofilin^S5A^* mutant cassette included two parts. One part was amplified from the parental cassette with primers cofS5A-upS and cofS5A-upA (including mutant site). The other part was amplified from the parental cassette with primers cofS5A-dwS (including mutant site) and cofS5A-dwA. Two parts were fused to construct *cofilin^S5A^* mutant cassette with primers cofS5A-upS and cofS5A-dwA. The cassette was transformed into CEA17*Δku80* protoplasts in the presence of PEG. The transformants were screened on AMM plates containing 1.2 M sorbitol and 0.1 μg ml^-1^ pyrithiamine.

### Construction of *Cofilin_teton_*/*Cofilin^S5E^ Strain*

Firstly, the *cofilin* gene including S5E mutation (*cofilin^S5E^*) was constructed by fusion PCR. One part of *cofilin^S5E^* sequence was amplified with the primer pair GFP-cofS5EWJ-upS and GFP-cofS5EWJ-upA from the *A. fumigatus* gDNA. The other part of *cofilin^S5E^* sequence was amplified with the primer pair GFP-cofS5EWJ-dwS and GFP-cofS5EWJ-dwA from the *g*DNA. Then the two parts were fused to form *cofilin^S5E^* sequence with primer pair GFP-cofS5EWJ-upS and GFP-cofS5EWJ-dwA. The *cofilin^S5E^* sequence was purified to clone into the EcoRV site of plasmid pJW103-hph-gpdA(p)-sGFP, forming the plasmid pLH2. Then the plasmid pLH2 was transformed into *cofilin_teton_* protoplasts to construct *cofilin_teton_*/*cofilin^S5E^* strain. The transformants were screened on AMM plates containing 1.2 M sorbitol, 100 μg ml^-1^ doxycycline and 200 μg ml^-1^ hygromycin.

### Morphological Characterization and Measurement of Mycelial Growth Rate

A total of 3 × 10^5^ conidia (3 μl) were inoculated centrally in AMM containing the doxycycline with the indicated concentration at 28, 37, and 48°C for 3 days. The colony morphology was observed and colony diameter was measured after 3 days, and the mycelial growth rate was determined as the increase in colony diameter per day (mm day^-1^). Notably, the max diameter of *cofilin_teton_* strain colony was measured. Radial growth tests were performed in triplicate for each strain.

### Stress Susceptibility Testing

For testing stress susceptibility among WT, *cofilin_teton_, cofilin^S5A^*, and *cofilin_teton_*/*cofilin^S5E^*, drop dilution assays were performed in a series of 10-fold dilutions derived from a starting suspension of 1 × 10^8^ conidia ml^-1^. Aliquots of 2 μl were spotted onto the indicated agar plates including various stresses (pH 5.0, pH 7.0, pH 9.0, H_2_O_2_, SDS, calcofluor white, Congo Red and farnesol) and cultured for 48 h at 37°C. To adjust the pH, media were supplemented with HCl or NaOH.

### RNA and cDNA Preparation

To detect expression of inflammatory factors (IL-8, MCP-1, and TNF-α), A549 cells (1 × 10^6^ per well) were seeded in 35 mm petri dishes and grown at 37°C, 5% CO_2_ for 18–24 h. When the conidia stimulated the cells directly, 1 ml fresh RPMI 1640 medium containing 1 × 10^7^ conidia and 3 μg ml^-1^ doxycycline was added into the well instead of the original 1640 medium and cultured for 6 h at 37°C, 5% CO_2_. Finally, discard the 1640 medium in 35 mm petri dishes and add 1 ml TRIzol^®^ Regeant (15596026, Invitrogen Life Technologies) for resuspending the cells. Total RNA was isolated using TRIzol^®^ Regeant according to the manufacturer’s instructions. First-strand cDNA synthesis was performed with an Anchored Oligo(dT)_18_ Primer using the EasyScript One-step gDNA Removal and cDNA Synthesis SuperMix (AE311-03, TransBionovo) according to the manufacturer’s instructions.

To detect gene expression of *A. fumigatus*, the conidia (4 × 10^7^) were inoculated into 40 ml AMM liquid medium supplemented with 3 μg ml^-1^ and 10 μg ml^-1^ doxycycline and cultured at 37°C, 200 rpm for 18 h. Mycelia were collected by gauze and frozen in liquid nitrogen. Then mycelia were ground to a powder and weighted 30–50 mg to resuspend in 1 ml TRIzol^®^ Regeant. RNA and cDNA preparation of the mycelia was same to the cells as described above.

### Quantitative Real-Time RT-PCR

For quantitative gene expression, a SYBR^®^ Premix Ex Taq^TM^ II (RR820A, Takara) and a Bio-Rad iQ5 real-time PCR system were used following the manufacturer’s instructions. Primers used for *A. fumigatus*-related genes are shown in Supplementary Table S2. Cycle conditions include two sections. One section for amplification is 3 min at 95°C and 40 cycles of 10 s at 95°C and 30 s at 55°C. The other section for melt curve is 1 min at 95°C, 1 min at 55°C followed by 55 to 95°C at 0.5°C s^-1^ melt rates. Relative quantification relates the PCR signal of the target transcript in a sample to control based on 2^-ΔΔCt^ method (Livak and Schmittgen, 2001). 18SrRNA was used as reference genes. Relative expression ratios were calculated by first calculating the cycle threshold changes in sample and control as Δ*Ct^Sample^*= *Ct*_(target)_-*Ct*_(reference)_ and Δ*Ct^control^*= *Ct*_(target)_-*Ct*_(reference)_ followed by calculating ΔΔ*Ct =* Δ*Ct^Sample^*-Δ*Ct^control^* and relative fold change = 2^-ΔΔ^*^Ct^*. Three replicates were performed per experiment.

### Protein Preparation and Western Blot

The conidia (4 × 10^7^) were inoculated into 1 ml AMM liquid medium including 3 μg ml^-1^ and 10 μg ml^-1^ doxycycline and cultured at 37°C, 180 rpm for 7.5 h followed by 100 μg ml^-1^ CFW stimulus. After the additional 40 min-incubation, mycelia were collected by centrifugation at 16,000 *g* for 10 min and resuspended in 200 μl protein extraction buffer [2% (w/v) SDS, 5% (v/v) mercaptoethanol, 60 mM Tris/HCl (pH 6.8), 10% (v/v) glycerol, 0.02 (w/v) bromophenol blue and protease inhibitor cocktail (CW2200S, Cwbitech)] (Dichtl et al., 2012). The suspension was immediately incubated on FastPrep-24^TM^ 5G (MP Biomedicals, United States) with a speed of 5.5 m s^-1^ for 40 s to extract the total proteins followed by heat denaturation at 100°C for 10 min. The supernatants were collected by centrifugation at 16,000 *g* for 10 min and served as the total cellular protein extracts for SDS-polyacrylamide gel electrophoresis (PAGE) as described previously (Bao et al., 2015). The concentration of total protein was balanced using the Histone H3 as a loading control. The self-casted SDS/15% polyacrylamide gels with 10 wells were 1.5 mm thick. A Mini-PROTEAN^®^ Tetra handcast system (Bio-Rad, United States) was used for protein electrophoresis and blotting. When the horseradish peroxidase (HRP) conjugated to second antibody reacted with Western Blot Luminol Reagent (sc-2048, Santa Cruz Biotechnology) in the PVDF membrane, the specific protein bands were visualized by autoradiography on Kodak X-ray film.

### Adherence Assay

The adherence capacity of *A. fumigatus* to epithelial cells was determined as described previously (Gravelat et al., 2010; Li et al., 2012). A549 cells were seeded in 6-well plates and grown for 24 h. The conidia (1.5 × 10^2^) of WT and *cofilin_teton_* were inoculated in 1 ml RPMI 1640 medium including 3 μg ml^-1^ doxycycline at 37°C for 8 h. Then the conidia suspensions were transferred into the 6-wll plates for 30 min at 37°C, followed by three washes with PBS including 0.1% Tween-20 to remove non-adherent fungi and overlaid with AMM agar supplemented with 100 μg ml^-1^ doxycycline. The number of adherent organisms was quantified by colony counting. Adherence was determined as the percentage of colonies related to the initial inoculum.

### Cell Wall Surface Analysis

Surface exposed β-1, 3-glucans were assayed by immunofluorescence with an antibody (400-2, Biosupplies). Briefly, 100 μl AMM liquid medium containing 1 × 10^7^ conidia and 3 μg ml^-1^ doxycycline was added in 96-well plate containing glass coverslips at 37°C, 5% CO_2_ for 6 h. After that, the conidia were fixed in 2.5% paraformaldehyde for 1 h at room temperature followed by three wash with PBS and blocked in 5% bovine serum albumin for 30 min. The conidia were then labeled with β-1, 3-glucan monoclonal antibody (100 μg ml^-1^) for overnight at 4°C and followed by three wash with PBS. Then tetraethyl rhodamine isothiocyanate (TRITC)-Conjugated Goat Anti-Mouse IgG (ZF-0313, ZSGB-BIO) was added into the 96-well plate in dark for 1 h. All procedures were at room temperature. Stained conidia were imaged with Olympus BX51 fluorescent microscope.

The glucosamine moiety of chitin/chitosan labeled with WGA-FITC (L4895, Sigma) was detected by flow cytometry (FCM). 1 ml AMM liquid medium containing 1 × 10^5^ conidia and 3 μg ml^-1^ doxycycline was added into 1.5 ml centrifuge tube and inoculated at 37°C, 5% CO_2_ for 6 h followed by addition of 2 μl Tween-20. Vortex seconds and centrifuge 15 min at 25°C, 20,000 *g*. Discard the supernatant and add 200 μl WGA-FITC (100 μg ml^-1^). Mix immediately by pipetting and keep the mixture from light at room temperature for 15 min. Wash once with 500 μl PBS and resuspend with 350 μl PBS followed by FCM detection.

### *In vitro* Internalization Assay

The rate of internalization of *A. fumigatus* by lung epithelial cells A549 was analyzed as described previously (Li et al., 2012). Briefly, human A549 cells were inoculated onto 96-well plates at a density of 2 × 10^4^ cells per well. Subsequently, 100 l AMM liquid medium containing 4 × 10^5^ conidia and 3 μg ml^-1^ doxycycline was added and incubated at 37°C under 5% CO_2_ to induce internalization. After 6 h internalization, the cell monolayers were washed three times with PBS, and 100 μl 1640 medium supplemented with 20 μg ml^-1^ nystatin was added to each well and incubated for 4 h to kill non-internalized conidia. The cell monolayers were then washed 3 times and treated with 100 l of PBS containing 0.25% Triton X-100 for 15 min at 37°C to induce cell lysis and the release of internalized conidia. The released conidia were diluted onto AMM plates supplemented with 100 μg ml^-1^ doxycycline and incubated at 37°C for 20 h. Colonies were counted to determine the total bound and intracellular conidia. The internalization rates were determined as the percentage of intracellular conidia colonies compared to the initial inoculum of conidia.

### *In vivo* Virulence Assay

The fifteen male BALB/c mice (body weight, 20–22 g) in each group were infected. Mice were immunosuppressed by hydrocortisone acetate. Each mouse was subcutaneous injection of 5 mg hydrocortisone acetate in 100 μl 0.1% PBST on days -4, -2, 0 and 10 mg hydrocortisone acetate on day 2. The *A. fumigatus* conidia used for infection should be fresh. The strains resuspended at a concentration of 5 × 10^6^ conidia ml^-1^. Mice were anesthetized by halothane inhalation and infected by intranasal instillation of 1 × 10^5^ conidia in 20 μl of 0.01% PBST. Mice were housed under sterile conditions and observed two times 1 day. The statistical significance of comparative survival values was calculated with Log-rank test using the GraphPad Prism 6.0 software.

### *G. mellonella* Infection Model

*Galleria mellonella* used for experiments are selected to be similar in size (approximately 0.3–0.5 g) and absent of any gray markings. Larvae were infected in groups of 16 with 5 × 10^5^ conidia resuspended in 10 μl 0.01% PBST per larva. The conidia suspension of *cofilin_teton_* and *cofilin_teton_*/*cofilin^S5E^* strain was additionally supplemented with 10 μg ml^-1^ doxycycline per larva. In each experiment, a group of 16 untreated larvae, a group of 16 larvae injected with 10 μl 0.01% PBST. Larvae were maintained in 9 cm Petri dishes at 37°C in the dark and examined every 12 h. The statistical significance of comparative survival values was calculated with Log-rank test using the GraphPad Prism 6.0 software.

### Statistical Analysis

Data shown in the figures are either from a representative experiment in triplicate or presented as mean ± standard error (SE) of 3∼4 independent experiments. Student’s unpaired *t*-test performed between two groups. Survival curves were analyzed using the Log-rank (Mantel-Cox) test. ^∗^*P* < 0.05 represents significantly different.

## Results

### Cofilin Is Essential for the Viability of *Aspergillus fumigatus*

**FIGURE 1:**
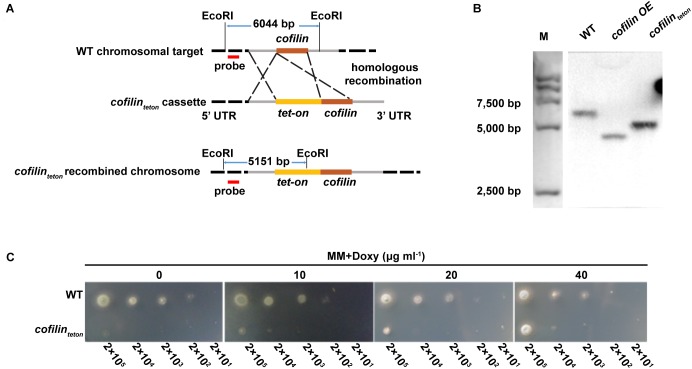
Construction and verification of *Aspergillus fumigatus* conditional *cofilin* deletion strain. **(A)** Schematic diagram of construction and Southern-blot verification of *cofilin_teton_*. A doxycycline-inducible promoter system (*tet-on*) was inserted into upstream of *cofilin* gene initiation codon by homologous recombination. The genomic DNA of two strains were digested with the restriction enzyme EcoRI and the digested products were hybridized with the digoxigenin-labeled probe (red). **(B)** After illumination, the specific probe bound to a 6,044 bp fragment in the WT, and a 5,151 bp fragment in *cofilin_teton_*. The positive control was a reported hybridization band of *cofilin OE*. **(C)** Aliquots of 2 μl WT and *cofilin_teton_* conidia of *A. fumigatus* in series of 10-fold dilutions derived from a starting suspension of 1 × 10^8^ conidia ml^-1^ were spotted on solid AMM medium supplemented with the indicated concentration of doxycycline and incubated for 30 h at 37°C.

A phylogenetic tree was deduced from alignment of the cofilin protein sequences of *A. fumigatus*, other fungi, mouse and *Homo sapiens* (Supplementary Figure S1). Cofilin (XP_753587.1) of *A. fumigatus* had rather distant homology with its counterpart in *Homo sapiens* (22%) and *Saccharomyces cerevisiae* (34%), respectively. To characterize the function of cofilin in *A. fumigatus*, initially we tried to construct two mutants including overexpression strain and null strain. The former has been reported in our recent publication (Jia et al., 2017), but the cofilin null strain was never successfully established. This led us to speculate that the *cofilin* gene was essential for the viability of *A. fumigatus*. Therefore, we generated a *cofilin_teton_* strain by replacing endogenous *cofilin* promoter of *A. fumigatus* CEA17*Δku80* wild-type (WT) strain with a doxycycline-controlled tet-on promoter (Figure 1A). The *cofilin_teton_* strain was verified by Southern blot (Figure 1B). As shown in Figure 1C, the *cofilin_teton_* strain was not able to grow on AMM medium lacking doxycycline, which indicated cofilin was indispensable for the viability of *A. fumigatus*. The growth of *cofilin_teton_* was rescued when the medium was supplemented with doxycycline and the growth rate increased along with the increased concentration of doxycycline. When the concentration of doxycycline reached 40 g ml^-1^, the *cofilin_teton_* strain grew more similar to the WT strain.

### Downregulation of Cofilin Affects Polarized Growth and Thermo-Tolerance of *A. fumigatus*

**FIGURE 2:**
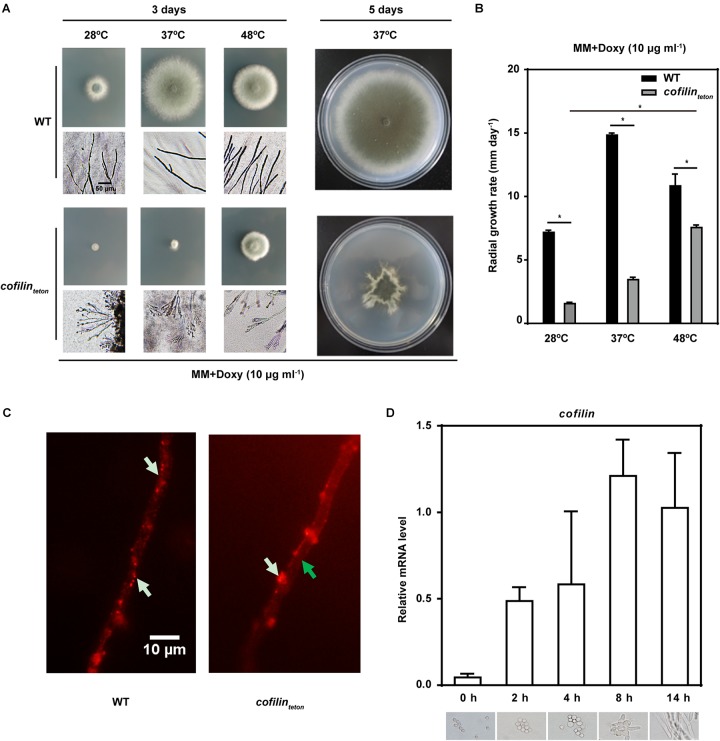
The morphology and growth of *cofilin_teton_* and WT. **(A)** 3 μl Conidia (1 × 10^8^ ml^-1^) of *cofilin_teton_* and WT were spotted in the center of solid AMM medium supplemented with doxycycline (10 μg ml^-1^) and incubated at 28°C, 37°C and 48°C, respectively. Left panel: representative picture of *A. fumigatus* colony cultured for 3 days and hyphae under normal light microscopy. Scale bar, 50 μm. Right panel: representative picture of *A. fumigatus* colony cultured for 5 days at 37°C. **(B)** 3 × 10^5^ Conidia were spotted in the center of solid AMM medium supplemented with the indicated concentration of doxycycline and incubated at 28, 37, and 48°C, respectively. The growth rates were determined as the increase in colony diameter per day (mm day^-1^). Data are represented as mean ± SE (*n* = 3). ^∗^*P <* 0.05. **(C)** The hyphal actin of *cofilin_teton_* and WT were examined under an Olympus fluorescent microscope. White arrow indicates actin. Green arrow indicates actin loss. Scale bar, 10 μm. **(D)** The wild-type strain was incubated in liquid AMM medium for the indicated time at 200 rpm and 37°C. Upper panel: the relative mRNA level of *cofilin* in different culture time was quantified by RT-qPCR. Data are represented as mean ± SE (*n* = 4). Lower panel: the morphology of strain at different time-point were observed under Olympus microscopy.

When *cofilin_teton_* was cultured in solid AMM containing lower concentration of doxycycline (10 μg ml^-1^), its hyphal tips were irregular and hyperbranched compared to WT, which indicated downregulation of cofilin impaired the polarized growth of *A. fumigatus* at different temperatures, 28, 37, 48°C (Figure 2A). The radial growth of *cofilin_teton_* cultured at 37°C for 5 days was also affected, which might have resulted from loss of hyphal polarity. The growth rate of *cofilin_teton_* was lower than WT at different temperatures, whereas the *cofilin_teton_* strain grew much faster at 48°C than at 28°C and 37°C (Figure 2B). Further, we also tested the effect of downregulation of cofilin on actin cytoskeleton in the hyphae. As illustrated in Figure 2C, actins (red-labeled) were relatively dispersed in the hyphae of WT, while they were reduced (green arrow) and aggregated in the cell wall (white arrow) of *cofilin_teton_*. We detected the expression of *cofilin* gene at different growth phases of *A. fumigatus*. The mRNA level of *cofilin* gene went up along the growth of *A. fumigatus* conidia with the peak at 8 h (Figure 2D), which was generally in accordance with conidial germination and formation of hyphae. All these data demonstrated that cofilin might be a critical factor for the polarized growth of *A. fumigatus*.

### Downregulation of Cofilin Affects Cell Wall Integrity Pathway in *A. fumigatus*

**FIGURE 3:**
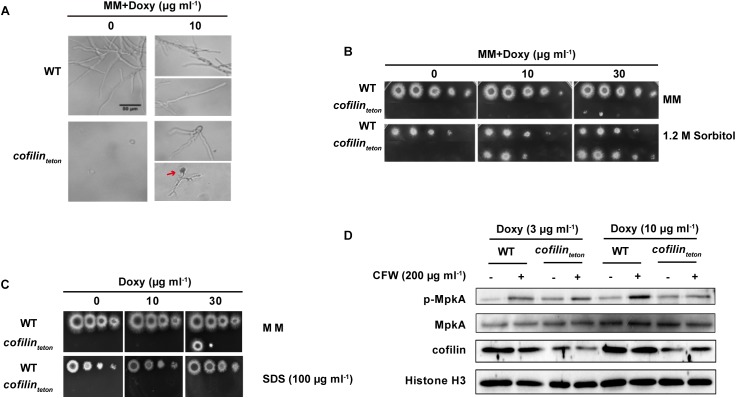
Effect of cofilin downregulation on polarized growth and cell wall integrity of *A. fumigatus*. **(A)** 5 × 10^3^ Conidia of WT and *cofilin_teton_* were inoculated in liquid AMM supplemented with the indicated doxycycline on glass coverslips and cultured at 37°C. Bright-field microscopy images were taken after the strains were fixed. The hyphal cytoplasmic leakage was indicated with red arrow. Scale bar, 50 μm. **(B)** In a series of 10-fold dilutions derived from a starting suspension of 1 × 10^8^ conidia ml^-1^ of the indicated strains, aliquots of 2 μl were spotted on AMM supplemented with the amount of doxycycline with or without 1.2 M sorbitol and incubated for 48 h at 37°C. **(C)** In a series of 10-fold dilutions derived from a starting suspension of 1 × 10^8^ conidia ml^-1^ of the indicated strains, aliquots of 2 μl were spotted on AMM supplemented with the amount of doxycycline with or without 100 μg ml^-1^ SDS and incubated for 48 h at 37°C. **(D)** The conidia of WT and *cofilin_teton_* were cultured in liquid AMM containing the indicated doxycycline at 180 rpm, 37°C for 7.5 h followed by stimulation with 200 μg ml^-1^ CFW for additional 40 min. Total proteins were extracted and the level of MpkA expression and phosphorylation were detected by western blot. Histone H3 was a loading control. Data are characteristic of 4 similar experiments.

When cultured in liquid AMM containing 10 μg ml^-1^ doxycycline, the *cofilin_teton_* strain displayed hyperbranched hyphal morphology and cytoplasmic leakage at hyphal tips (red arrow indicated in Figure 3A). This data confirmed that downregulation of cofilin severely impaired the growth polarity and this defect could be rescued by supplementation of 1.2 M sorbitol (Figure 3B). Since the cytoplasmic leakage indicated that cofilin might be closely involved in regulation of CWI of *A. fumigatus* (Dichtl et al., 2012), the sensitivity of *cofilin_teton_* to several cell wall perturbing agents was investigated. Downregulation of cofilin could increase the sensitivity of *A. fumigatus* to SDS (Figure 3C), but not other three classical cell wall perturbing agents, calcofluor white (CFW), farnesol (FOH), Congo Red (CR) (Supplementary Figure S2). It’s well known that CWI signaling cascade in *A. fumigatus* is central to sense a wide range of extracellular stress to orchestrate the cellular response and related to virulence (Valiante et al., 2015). And the kinase MpkA is the core signaling protein in the CWI pathway (Li et al., 2011). To determine whether cofilin was able to regulate the classical MpkA-dependent CWI pathway in *A. fumigatus*, the phosphorylation of MpkA was detected by Western blot and it was found that under normal condition the basal phosphorylation of MpkA increased significantly in *cofilin_teton_* compared to WT (Figure 3D). CFW-induced MpkA phosphorylation was similar in WT and *cofilin_teton_* cultured with 3 μg ml^-1^ doxycycline, but CFW-induced MpkA phosphorylation was reduced in *cofilin_teton_* compared with WT cultured with 10 μg ml^-1^ doxycycline.

### Downregulation of Cofilin Increased the Sensitivity of *A. fumigatus* to Alkaline pH and Oxidative Stresses

**FIGURE 4:**
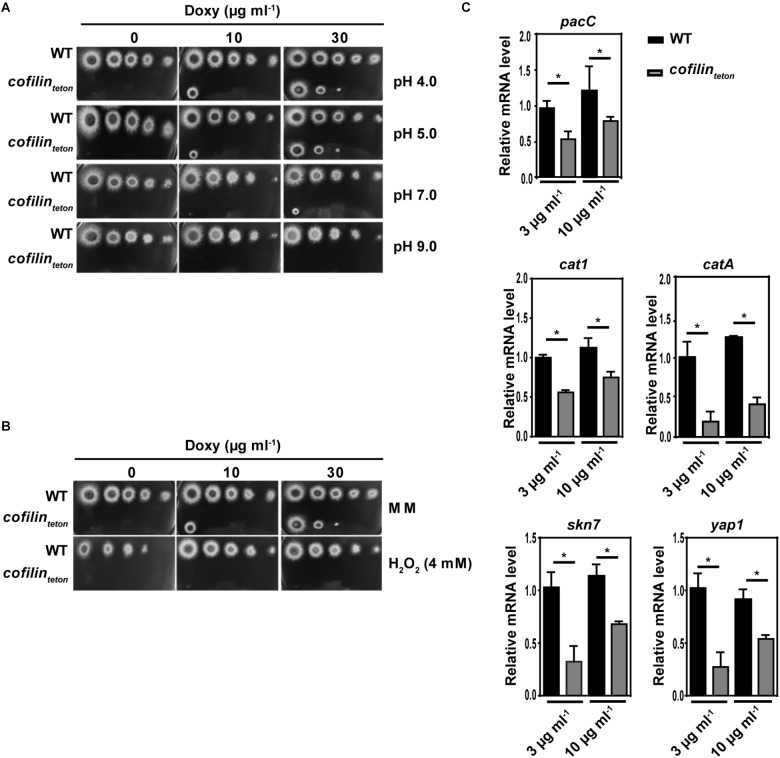
Role of cofilin on alkaline pH and oxidative stress response of *A. fumigatus*. **(A)** In a series of 10**-**fold dilutions derived from a starting suspension of 1 × 10^8^ conidia ml^-1^ of the indicated strains, aliquots of 2 μl were spotted on AMM containing the amount of doxycycline at different pH values. **(B)** In a series of 10**-**fold dilutions derived from a starting suspension of 1 × 10^8^ conidia ml^-1^ of *cofilin_teton_* and WT, aliquots of 2 μl were spotted on AMM containing the amount of doxycycline with or without 4 mM H_2_O_2_. **A,B**: after a 48 h incubation at 37°C, the colony growth was comparatively analyzed. **(C)** The conidia of WT and *cofilin_teton_* were cultivated in liquid AMM containing doxycycline at concentrations of 3 and 10 μg ml^-1^ for 18 h. The mRNA expression levels of *cofilin* gene and oxidative stress-related genes were tested by RT-qPCR. Data are represented as mean ± SE (*n* = 3). ^∗^*P <* 0.05.

We further studied whether downregulation of cofilin could affect pH and oxidative response of *A. fumigatus*. Along with the increase of pH value from 4.0 to 9.0, the *cofilin_teton_* strain became more susceptible than WT (Figure 4A). Compared with WT, the *cofilin_teton_* also showed significantly increased sensitivity to 4 mM H_2_O_2_ (Figure 4B). Further, expression of several critical genes (*pacC, catA, cat1, skn7* and *yap1*) associated with response to alkaline pH and oxidative stress in *A. fumigatus* was detected by RT-qPCR. In Figure 4C, it was shown that mRNA levels of these genes were significantly lower in *cofilin_teton_* compared with WT. The stress-related genes of the *cofilin_teton_* strain showed elevated expression levels as the doxycycline concentration increased from 3 to 10 μg ml^-1^. Taken together, these data demonstrated that cofilin had an important role in alkaline pH and oxidative stresses of *A. fumigatus*.

### Downregulation of Cofilin Alters the Polysaccharide Composition in the Cell Wall and Impairs the Pathogenicity of *A. fumigatus*

**FIGURE 5:**
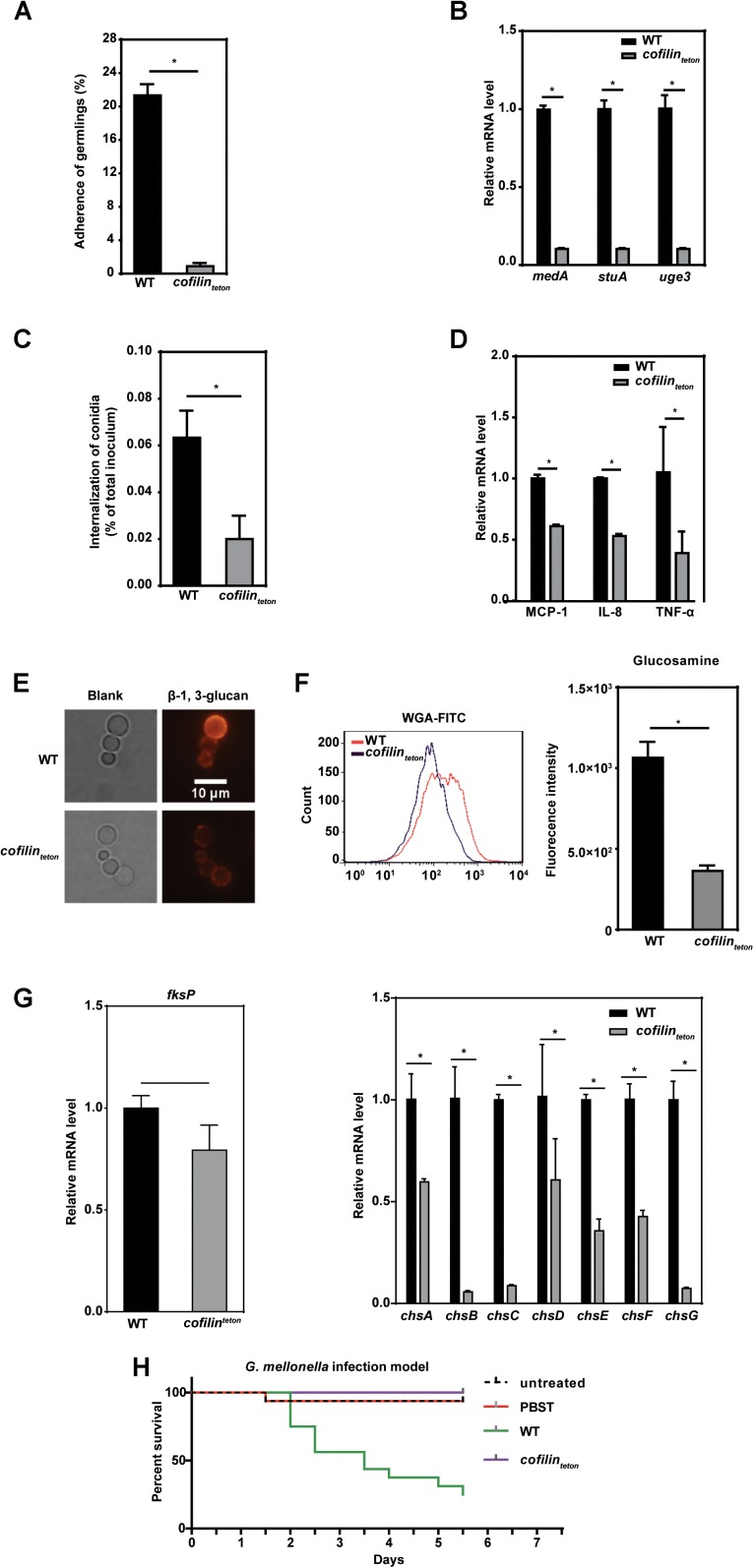
Effect of cofilin downregulation on pathogenicity and cell wall polysaccharide synthesis of *A. fumigatus*. **(A)** Adherence of the WT and *cofilin_teton_* (1.5 × 10^2^) at the similar germinating phase to A549 cells was measured. **(B)** Expression of three adherence-related genes of *A. fumigatus, medA, stuA* and *uge3*, was detected by RT-qPCR. **(C)** 2 × 10^4^ A549 cells were infected with the resting conidia of the indicated strains at an MOI of 20 at 37°C for 6 h. The internalization of *A. fumigatus* to host cells were analyzed by the nystatin protection assay. **(D)** The conidia from *cofilin_teton_* were inoculated into 1 × 10^6^ A549 cells at an MOI of 10 and co-cultured at 37°C for 6 h. Thereafter, the expression level of inflammatory factors MCP-1, TNF-α and IL-8 was detected by RT-qPCR. **A–D:** data are represented as mean ± SE (*n* = 3–4). ^∗^*P <* 0.05. **(E)** The indicated conidia cultured at 37°C for 6 h in liquid AMM medium were labeled with anti-β-1, 3-glucan antibody and detected with an Olympus fluorescent microscope. The red color indicates β-1, 3-glucan on the cell wall of *A. fumigatus*. Scale bar, 10 μm. **(F)** The glucosamine moiety of chitin/chitosan in the indicated conidia was labeled with WGA-FITC and detected with flow cytometry. Left panel: flow cytometry diagram in one representative experiment. Right panel: histograms represented the difference of fluorescence intensity among *cofilin_teton_* and WT strains. **(G)** Expression of β-1, 3-glucan synthetas *fksP*, chitin synthetase family genes in WT and *cofilin_teton_* strains was detected by RT-qPCR. Data are represented as mean ± SE (*n* = 3 - 4). ^∗^*P <* 0.05 **(H)** In *G. mellonella* infection model, larvae were infected in groups of 16 with 5 × 10^5^ conidia resuspended in 10 μl 0.01% PBST per larva. The conidia suspensions of *cofilin_teton_* strain were additionally supplemented with doxycycline (10 μg ml^-1^ per larva). The statistical significance of comparative survival values was calculated with Log-rank test using the GraphPad Prism 6.0 software. The results shown are representative of 3 experiments.

It’s well known that colonization and invasion of *A. fumigatus* into lung epithelial cells are important for the dissemination of *A. fumigatus* infection (Murayama et al., 1996). To assess the possible role of *A. fumigatus cofilin* on these processes, we tested the adherence and internalization of *cofilin_teton_* and WT strains to human lung epithelial cells. Compared to WT, *cofilin_teton_* adhered much less to lung epithelial A549 cells (Figure 5A). Since several genes including *medA, stuA and uge3* are known to be associated with the adherence of *A. fumigatus* to host cells (Al Abdallah et al., 2012; Lin et al., 2015), it is interesting to test whether downregulation of cofilin affected the transcription of these genes. By RT-PCR, it was found that mRNA levels of the three genes, *medA, stuA and uge3* in *cofilin_teton_* were significantly reduced to 15% of WT, which was in line with the decreased adherence (Figure 5B). Similarly, the internalization rate of *cofilin_teton_* conidia into lung epithelial A549 cells was also significantly lower than that of WT (Figure 5C). Three inflammatory factors including MCP-1, IL-8, TNF-α released by A549 cells were also detected during interaction between host cell and *A. fumigatus*. The levels of these three factors induced by *cofilin_teton_* were much lower than WT (Figure 5D). Since it has been reported that cell wall polysaccharides influenced internalization of *A. fumigatus* and inflammatory response of host cell (Jia et al., 2017), we further found the β-1, 3-glucan (red fluorescence labeled) on the cell wall of *cofilin_teton_* were significantly lower than that of WT (Figure 5E). And the glucosamine moiety of chitin/chitosan in the cell wall of *cofilin_teton_* decreased as well (Figure 5F). Next, the mRNA levels of several genes encoding key synthases of β-1, 3-glucan and chitin/chitosan were measured. As shown in Figure 5G, the mRNA levels of β-1, 3-glucan synthetase (*fksP*) and chitin synthetases (*chsA, chsB, chsC, chsE, chsF, chsG*) in *cofilin_teton_* mutant were significantly lower.

Finally, to further characterize the possible effect of cofilin on pathogenicity of *A. fumigatus*, an *Galleria mellonella* model which had been demonstrated as a good model to evaluate fungal pathogenicity was used (Slater et al., 2011). As shown in Figure 5H, the mortality of worms infected by *cofilin_teton_* was far lower than those infected by WT. All these data indicated that cofilin might be involved in the regulation of polysaccharide composition of cell wall, and also the interaction of *A. fumigatus* with lung epithelial cells, which might affect the pathogenicity of *A. fumigatus*.

### Phosphorylation of Cofilin Is Critical for Hyphal Growth, MpkA Activation and Internalization of *A. fumigatus*

**FIGURE 6:**
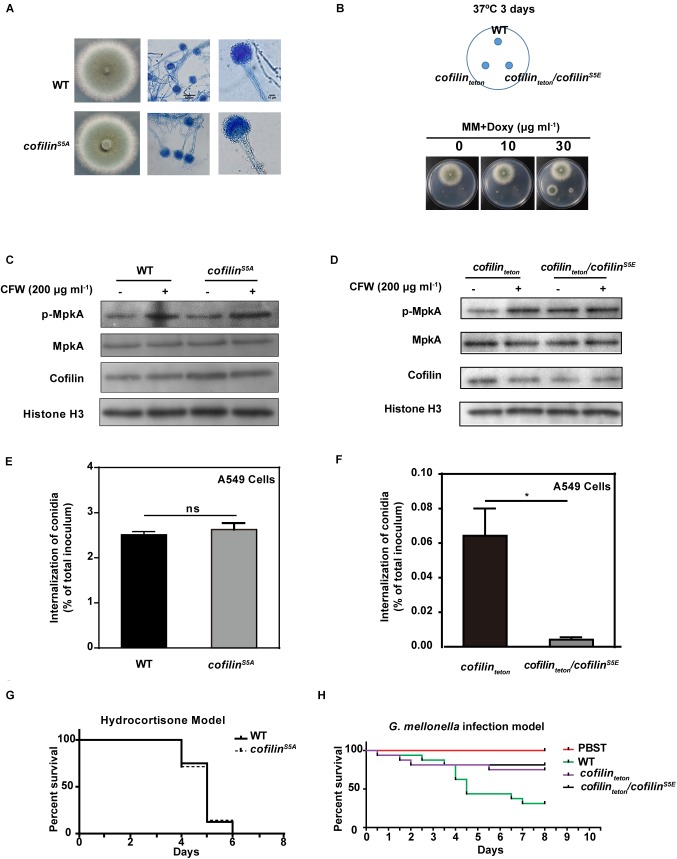
Effect of cofilin phosphorylation on the growth, stress response and virulence of *A. fumigatus*. **(A)** 3 × 10^5^ conidia of *cofilin^S5A^* and WT were spotted in the center of solid AMM medium and incubated at 37°C for 3 days. **(B)** 3 × 10^5^ conidia of WT or *cofilin_teton_* or *cofilin_teton_*/*cofilin^S5E^* were spotted at the indicated points on solid AMM medium supplemented with the doxycycline at different concentration and incubated at 37°C for 3 days. **A,B**: The morphologies of colony and hyphae were captured. The conidia of WT and *cofilin^S5A^* **(C)**, *cofilin_teton_* and *cofilin_teton_/cofilin^S5E^* **(D)** were cultured at 180 rpm, 37°C for 7.5 h followed by stimulation with 200 μg ml^-1^ CFW for additional 40 min. Total proteins were extracted and the expression and phosphorylation of MpkA protein was detected by western-blot. **C,D**: the results shown are representative of 4 experiments. 4 × 10^5^ Conidia of WT and *cofilin^S5A^* **(E)**, *cofilin_teton_* and *cofilin_teton_/cofilin^S5E^* **(F)** was inoculated and co-cultivated with 2 × 10^4^ A549 cells in RPMI 1640 medium for 6 h. The internalization of *A. fumigatus* into A549 cells was analyzed by the nystatin protection assay. **E,F**: Data are represented as mean ± SE (*n* = 3–4). **(G)** The survival values of the hydrocortisone immunocompromised murine infected with WT and *cofilin^S5A^*. **(H)** The survival values of *G. mellonella* larvae infected with WT, *cofilin_teton_ and cofilin_teton_/cofilin^S5E^*. **G,H**: the statistical significance of comparative survival values was calculated with Log-rank test using the GraphPad Prism 6.0 software. The results shown are representative of 3 experiments. ^∗^*P <* 0.05.

As phosphorylation of cofilin is the key molecular switch to its function in actin cytoskeleton dynamic of mammalian cells, we investigated further the role of cofilin phosphorylation on the growth phenotype, cell wall composition, stress response and pathogenicity of *A. fumigatus*. Firstly, we determined the fifth serine (Ser5) at the N-terminal of cofilin was the phosphorylated residue in *A. fumigatus* through homology analysis. Then we planned to construct two mutants including cofilin^S5A^ (a non-phosphorylated form) and cofilin^S5E^ (a mimic phosphorylated form). However, the *cofilin^S5E^* mutant was not viable. We changed the initial strategy of S5E mutation at native locus of CEA17*Δku80* genome and constructed a *cofilin_teton_*/*cofilin^S5E^* strain by transforming a plasmid expressing GFP-fused cofilin^S5E^ (pLH2) into *cofilin_teton_* mutant. The *cofilin^S5A^* mutant cultured on AMM and SDA plates had the same morphology and growth rate as its parental strain at any temperature (Figure 6A and Supplementary Figure S4). Both *cofilin_teton_* and *cofilin_teton_*/*cofilin^S5E^* were not able to grow on AMM medium without doxycycline. The growth of *cofilin_teton_* could be rescued by supplement of doxycycline, but the additional cofilin^S5E^ expression could obviously blocked this rescue. As shown in Figure 6B, the colony of *cofilin_teton_*/*cofilin^S5E^* mutant grew much smaller than *cofilin_teton_* mutant.

The effect of cofilin phosphorylation on the stress responses of *A. fumigatus* was also evaluated. The sensitivity of *cofilin^S5A^* and *cofilin_teton_*/*cofilin^S5E^* mutants to cell-wall perturbing agents, H_2_O_2_ and alkaline pH was not altered compared to their parental strains, respectively (Supplementary Figure S5). Further, the phosphorylation of MpkA was detected in *cofilin^S5A^* and *cofilin_teton_*/*cofilin^S5E^* mutants. No significant alteration on MpkA phosphorylation between *cofilin^S5A^* and WT with or without CFW-stimulation was found (Figure 6C). The basal phosphorylation of MpkA in *cofilin_teton_*/*cofilin^S5E^* mutant without CFW-stimulation was even higher than *cofilin_teton_* mutant. Whereas no difference of CFW-induced MpkA phosphorylation was found between *cofilin_teton_*/*cofilin^S5E^* and *cofilin_teton_* mutants (Figure 6D).

Finally, internalization of *cofilin^S5A^* mutant and WT by A549 cells was similar (Figure 6E). In contrast, the internalized *cofilin_teton_*/*cofilin^S5E^* conidia were much less than *cofilin_teton_* conidia (Figure 6F). *In vivo*, no significant difference in survival rate of hydrocortisone-immunosuppressed mice infected by *cofilin^S5A^* and WT was found (Figure 6G). The *cofilin_teton_*/*cofilin^S5E^* and *cofilin_teton_* mutants in *G. mellonella* model also demonstrated similar virulence (Figure 6H).

## Discussion

In this study, we further investigated the function of *A. fumigatus* cofilin in more detail by constructing three mutants including *cofilin_teton_, cofilin^S5A^* and *cofilin_teton_*/*cofilin^S5E^*. First, it was confirmed that cofilin was essential for viability of *A. fumigatus* because *cofilin_teton_* could not grow without doxycycline. Downregulation of cofilin severely impaired growth rate and polarity of *A. fumigatus*. The hyphae of *cofilin_teton_* in both solid and liquid AMM were hyperbranched, which was similar to the null strains of *shol* and *myoE* in *A. fumigatus*. Since the transportation of components for polarized growth was relayed on actin cytoskeleton (Yang et al., 2011; Renshaw et al., 2016), the reduction of actin cytoskeleton in *cofilin_teton_* might disorder the trafficking and impair its polarity. Besides, mRNA level of *cofilin* gene was the highest at 8 h during the germinating phase of conidia, which further supported a close relationship of cofilin with polarized growth of *A. fumigatus*. Differently, the morphology of *cofilin OE* was similar to WT, and the polarity of *A. fumigatus* wasn’t influenced by cofilin overexpression.

The stress response of *cofilin_teton_* to several cell wall perturbing agents seemed in a line with the influence of overexpression of cofilin. First, both downregulation and overexpression of cofilin had no effect on the response of *A. fumigatus* to three classical cell wall perturbing agents, CFW, CR and FOH. Second, *cofilin_teton_* was more sensitive to SDS whereas *cofilin OE* had more resistance to SDS. SDS could be used as a cell wall stressor, but it mainly acts on cell membrane. Besides, downregulation of cofilin caused decreased heat sensitivity and increased constitutive MpkA phosphorylation. As a cell wall perturbing condition, heat stress is also regulated by CWI pathway (Dichtl et al., 2016). It can be deduced that cofilin might regulate the CWI pathway and cell membrane integrity from these results. Similar results have been reported in *A. fumigatus kexB* gene study. Deletion of *kexB* (encoding a subtilisin-like serine proteinase) also led to impaired CWI, abnormal polarity and activation of the basal MpkA phosphorylation (Wang et al., 2015). The susceptibility of Δ*kexB* mutant to CFW, FOH and CR was clearly raised compared to WT, which was quite different from *cofilin_teton_*. But the *cofilin_teton_* mutant and Δ*kexB* were thermotolerant at 48°C. These two results reflected that increased basal MpkA phosphorylation and CWI defect might produce different phenotypes in *A. fumigatus*. One study might provide a reference for exploring the possible relationship between cofilin and MpkA cascade. It has been reported that treatment of either rapamycin or latrunculin B which depolarizes the actin cytoskeleton could induce Mpk1 (a homology protein of MpkA) activation in *Saccharomyces cerevisiae* (Levin, 2005). Given that, we speculated that downregulation of cofilin might induce MpkA phosphorylation by impairing the homeostasis of actin cytoskeleton. Certainly, further investigation are needed for direct evidence.

Downregulation of cofilin resulted in increased sensitivity to alkaline pH and less transcription of *pacC*. However, it has been shown that pH is unable to affect cofilin activity in yeast (Bernstein and Bamburg, 2010). This indicated that cofilin had a different role in pH-induced signaling pathway of *A. fumigatus* and yeast. A good consistency on oxidative response of *cofilin OE* and *cofilin_teton_* was demonstrated. Downregulation of cofilin resulted in significant elevated susceptibility of *A. fumigatus* to H_2_O_2_. This might be associated with the decreased expression of oxidative-associated genes including *cat1, catA, skn7* and *yap1* (Figure 4C). However, it could not be excluded that the leaky membranes of *cofilin_teton_* might cause H_2_O_2_ to have a higher influx and lethal damage. And the lower expression of oxidative-associated genes in *cofilin_teton_* might result from lower metabolic activity and/or lower growth rate of the mutant. Besides, *cofilin_teton_* was also hypersensitive to a disruptor of ER homeostasis, dithiothreitol (DTT) (Richie et al., 2009) (data not shown). In consideration with the adverse effect of oxidative stress on ER homeostasis (Malhotra and Kaufman, 2007), cofilin might be also involved in regulation of ER stress in *A. fumigatus*, which needs further study.

It was interesting that cofilin expression was correlated with the alteration of cell wall composition of *A. fumigatus*, which might be a contributor to the lower internalization and inflammatory response in host cells (Bertuzzi et al., 2014; Lu et al., 2018). However, some other reasons for this lower internalization and less inflammatory response of *cofilin_teton_* mutant could not be excluded. Importantly, the different growth/germination profiles between WT and *cofilin_teton_* might be a critical confounding factor. Similarly to our results, the evidence of PacC-governed epithelial entry during pulmonary *Aspergillosis* also came from the comparison between WT and its null mutant (Δ*pacC*) that grows more slowly than WT cultured for the same hours (Bertuzzi et al., 2014). More, the metabolic change or leakage of potential toxic factors caused by lack of cofilin in *A. fumigatus* should be taken in consideration. Because some toxins (e.g., gliotoxin) are secreted into extracellular environment to promote internalization of *A. fumigatus* (Jia et al., 2014). In addition, the increased survival rates of *G. mellonella* infected by *cofilin_teton_* and *cofilin_teton_*/*cofilin^S5E^* indicated that cofilin played some role in pathogenicity of *A. fumigatus*. However, the *cofilin OE* had no impact on *G. mellonella* survival. So more investigations are needed to elucidate the exact role of cofilin in the interaction between *A. fumigatus* and host cells.

Another interesting finding was that the non-phosphorylated cofilin mutation (S5A), like overexpression of cofilin, did not have significant influence on phenotype, CWI and pathogenicity of *A. fumigatus*. In contrast, mimic-phosphorylated cofilin mutation (S5E) was lethal to *A. fumigatus*. It has been well known that the balance of phospho-cycle at serine 3 of cofilin in mammalian cells is indispensable to regulate uptake of pathogens. Expression of either cofilin S3A (non-phosphorylated form) or S3E (mimic-phosphorylated form) reduced *Listeria* internalization into Vero cells, while overexpression of wild-type cofilin and cofilin S3A mutation in A549 cells inhibited the internalization of *A. fumigatus*. These hinted cofilin in *A. fumigatus* and mammalian animal had some distinct functional mechanisms, which is probably attributed to the relative distant genetic relationship between them.

## Conclusion

This study showed for the first time that cofilin is essential for viability of *A. fumigatus*. Either downregulation or over-phosphorylation of cofilin affected the polarized growth, MpkA activation, stress response of *A. fumigatus* severely. If cofilin became non-phosphorylated form completely, there was little effect on *A. fumigatus*.

## Ethics Statement

This study was carried out in accordance with the recommendations of the Guide for the Care and Use of Laboratory Animals of the Ministry of Science and Technology of the People’s Republic of China, Laboratory Animal Welfare and Ethics Committee of Academy of Military Medical Sciences (license number IACUC-13-2016-002). The protocol was approved by the Laboratory Animal Welfare and Ethics Committee of Academy of Military Medical Sciences.

## Author Contributions

XJ and LH planned experiments and analyzed data. XJ, XZ, MH, and YH performed experiments. XH and YS contributed reagents or other essential material. XJ and LH wrote the paper.

## Conflict of Interest Statement

The authors declare that the research was conducted in the absence of any commercial or financial relationships that could be construed as a potential conflict of interest.

## Footnotes

1 http://www.ncbi.nlm.nih.gov/protein/
