# Supplementary material for: Role of Downregulation and Phosphorylation of Cofilin in Polarized Growth, MpkA Activation and Stress Response of Aspergillus fumigatus
Source: Front Microbiol. 2018 Nov 2;9:2667. doi: 10.3389/fmicb.2018.02667 (PMC6230985; doi:10.3389/fmicb.2018.02667)
Supplement: Supplementary file 1 [file Data_Sheet_1.PDF]

Supplementary Material

Role of Downregulation and Phosphorylation of Cofilin in Polarized Growth, MpkA Activation and Stress Response of *Aspergillus fumigatus*

Xiaodong Jia[#], Xi Zhang[#], Yingsong Hu, Mandong Hu, Xuelin Han, Yansong Sun[*], Li Han[*]

***Correspondence:**

Li Han, hanlicdc@163.com

Yansong Sun, sunys1964@hotmail.com

1. Supplementary Tables

Table S1. ***Aspergillus fumigatus*** **strains used in this study.**

Strain	Genotype	Reference
WT	*ΔKU80*	da Silva Ferreira, Kress et al. 2006
$cofilin_{teton}$	*ΔKU80, cofilin(p)::ptrA-tetOn::cofilin*	This study
$cofilin^{S5A}$	*ΔKU80, cofilin(p)::cofilin^{S5A}::ptrA*	This study
$cofilin_{teton}/cofilin^{S5E}$	*ΔKU80, cofilin(p)::ptrA-tetOn::cofilin*; pLH2	This study

Table S2. Primers used in this study.

No.	Primer	Sequence (5'-3')
1	coftj-upS	TTTACGCCTGTGAGCCACTTC
2	coftj-upA	ACAAAGATGCAAGAGGCCATCTCCCGAGGCTGATTTAGAAGATG
3	coftj-tetonS	CATCTTCTAAATCAGCCTCGGGAGATGGCCTCTTGCATCTTTGT
4	coftj-tetonA	CGGAATAATTATGACATACCGACATAGGCCGGTGATGTCTGCTC
5	coftj-dwS	GAGCAGACATCACCGGCCTATGTCGGTATGTCATAATTATTCCG
6	coftj-dwA	GAAGACAGACGACGTTTGACTGTG
7	coftj-yS	CTCATTTCCTTCGCTGTCG
8	coftj-yA	CAGATTGCTGGTGTTGTCGT
9	cof-southern-proS	ACCATGAGAACCGAGTTTGG
10	cof-southern-proA	CGAGTCCGCTGCATATTGTAC
11	*18SrRNA*-mS	TGAGCCGATAGTCCCCCTAA
12	*18SrRNA*-mA	GACTCAACACGGGGAAACTC
13	*cofilin*-mS	GGTAATCCCGCTCCTCGTT
14	*cofilin*-mA	GCGTAGATCATGGACCACAGA
15	*medA*-mS	TATTATGGGCTTTCCCGCCC

16	*medA*-mA	AGCTTGCAGAGTATTTTCCAATGA
17	*stuA*-mS	CCTTTACCCGTCAACAGCC
18	*stuA*-mA	GGTATCATAGACGAACCCTTGC
19	*uge3*-mS	GGGAGTGAACGCATTGTCC
20	*uge3*-mA	CCTCGCAGATCCATTTGGT
21	*cat1*-mS	CGGAGTGGGAACTCGGTG
22	*cat1*-mA	TGGCACGATCTTTGTGGG
23	*catA*-mS	GTACGAGTCAGCAACACCGA
24	*catA*-mA	GCGATGGATCTTTTCTCTGGC
25	*skn7*-mS	TGGACAAAACGCATGGGAGT
26	*skn7*-mA	TCTGTTGTGTCGGAACGGAG
27	*yap1*-mS	GCGATGAGAAAGCAGCCAAG
28	*yap1*-mA	TGCGCTTCGAAGTAGGTTCC
29	*pacC*-mS	TCAAGCGTCCCCAGGATCTA
30	*pacC*-mA	GCAGCATAGCCTTTTCCAGC
31	GAPDH-mS	GCCCTCAACGACCACTTTGT
32	GAPDH-mA	TGGTGGTCCAGGGGTCTTAC
33	MCP-1-mS	CGCCTCCAGCATGAAAGTCT
34	MCP-1-mA	AGGTGACTGGGGCATTGATTG
35	IL-8-mS	TGCCAGCTGTGTTGGTAGTG
36	IL-8-mA	TGACTGTGGAGTTTTGGCTGT
37	TNF-α-mS	CCCAGGGACCTCTCTCTAATCA
38	TNF-α-mA	GCTACAGGCTTGTCACTCGG
39	cofsite-m-upS	CACTCATTTCCTTCGCTGTCG
40	cofsite-m-upA	GTCGTTGCGTCAGTCCAACCATCGAGTCGGTCGCTTG
41	cofsite-m-ptrAs	CAAGCGACCGACTCGATGGTTGGACTGACGCAACGAC
42	cofsite-m-ptrAa	CGCAATAAGATGGTACGGTCACCACTTTATGCTTCCGGCTC
43	cofsite-m-dwS	GAGCCGGAAGCATAAAGTGGTGACCGTACCATCTTATTGCG
44	cofsite-m-dwA	ACTGCCTACCCAGCGTGTCT
45	cofS5A-upS	TTCCCTCCTGGCTCCTGTCA
46	cofS5A-upA	CAACATACCCTGCTGCGAGCTGCGAGGCAAGAAATACCATTAGT
47	cofS5A-dwS	ACTAATGGTATTTCTTGCCTCGCAGCTCGCAGCAGGGTATGTTG
48	cofS5A-dwA	TGGCGAACAGATGGATCACG
49	cofsite-m-seqS	CCTCTATCTCCATCTCCGTTTC
50	cofsite-m-seqA	CAATGTCGCCCTTGTCGT
51	GFP-cofS5EWJ-upS	AGGCGGCATGTCGGTATGTCATAATTATTCCG
52	GFP-cofS5EWJ-upA	CAAAAGGTATAAACGTCAACATACCCCTCTGCGAGCTGCGAGGCAAG
53	GFP-cofS5EWJ-dwS	CTTGCCTCGCAGCTCGCAGAGGGGTATGTTGACGTTTATACCTTTTG
54	GFP-cofS5EWJ-dwA	CTATTTACCGGCCTTACCACCG
