# Supplementary material for: Role of Downregulation and Phosphorylation of Cofilin in Polarized Growth, MpkA Activation and Stress Response of Aspergillus fumigatus
Source: Front Microbiol. 2018 Nov 2;9:2667. doi: 10.3389/fmicb.2018.02667 (PMC6230985; doi:10.3389/fmicb.2018.02667)
Supplement: Supplementary file 2 [file Data_Sheet_2.PDF]

Supplementary Material

Role of Downregulation and Phosphorylation of Cofilin in Polarized Growth, MpkA Activation and Stress Response of *Aspergillus fumigatus*

Xiaodong Jia[#], Xi Zhang[#], Yingsong Hu, Mandong Hu, Xuelin Han, Yansong Sun[*], Li Han[*]

***Correspondence:**

Li Han, hanlicdc@163.com

Yansong Sun, sunys1964@hotmail.com

1. Supplementary Figures

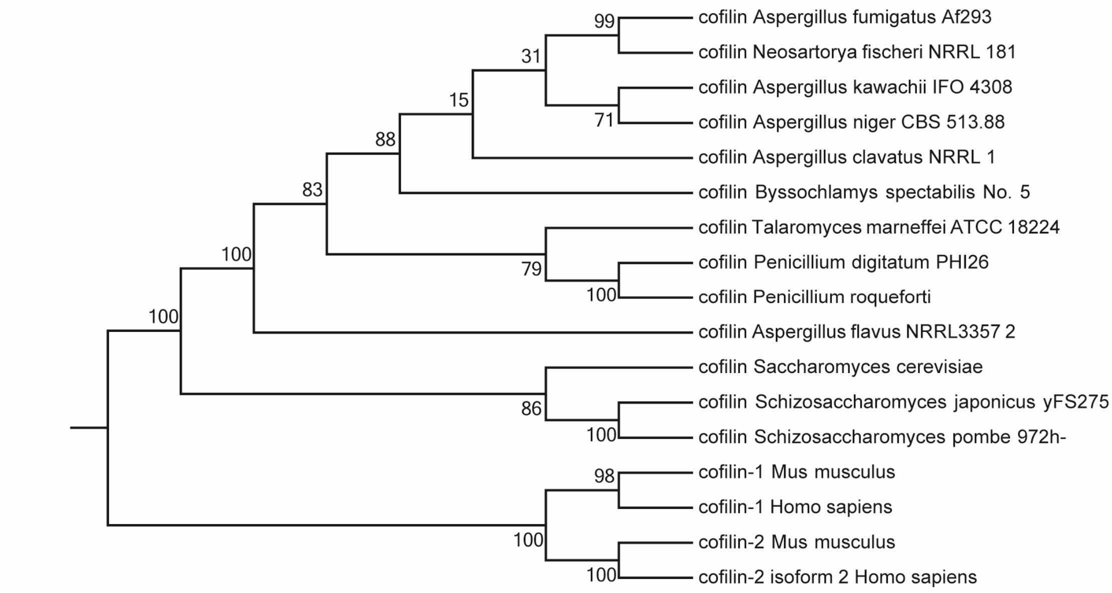

Figure S1. Phylogenetic tree of cofilin protein.

The map was constructed using MEGA 5.0 software.

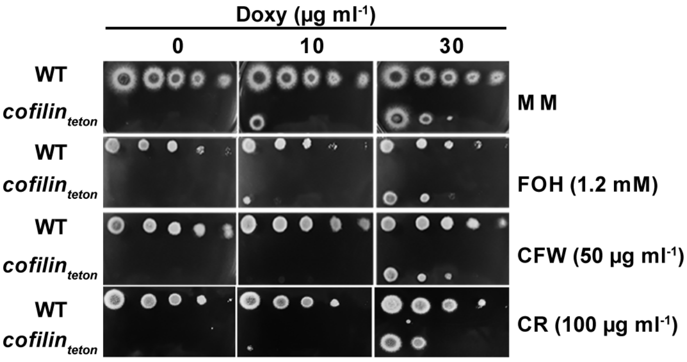

Figure S2. Sensitivity of *cofilin*$_{teton}$ to CFW, CR and FOH.

In a series of 10-fold dilutions derived from a starting suspension of 1×10^{8} conidia ml^{-1} of *cofilin*$_{teton}$ and WT, aliquots of 2 μl were spotted on AMM containing the indicated amount of doxycycline with or without 1.2 mM FOH, 50 μg ml^{-1} CFW and 100μg ml^{-1} CR.

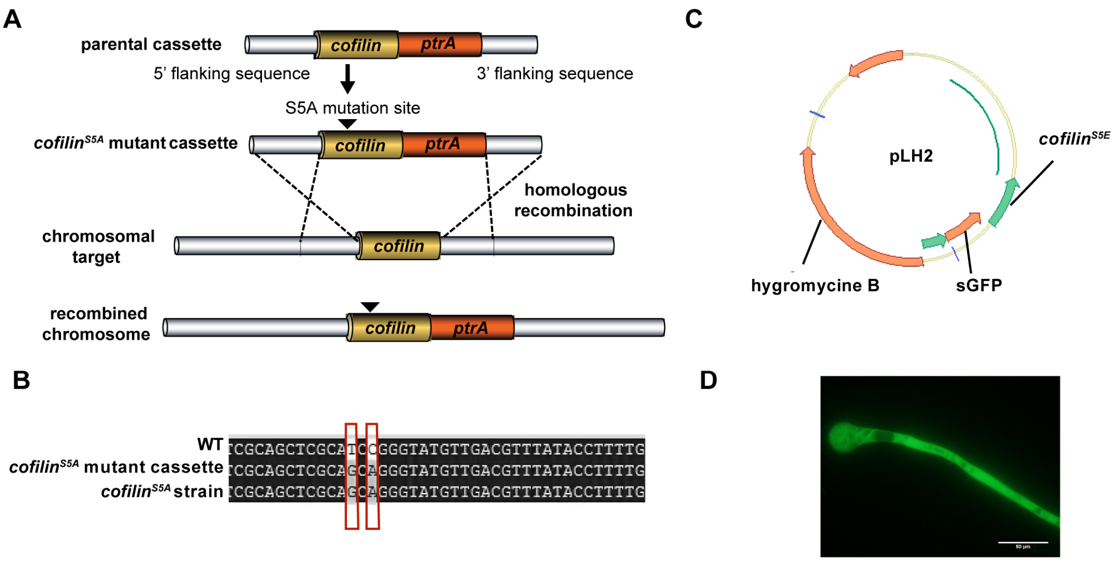

Figure S3. Schematic diagrams of construction and verification of *cofilin*^{S5A} and *cofilin*$_{teton}$/*cofilin*^{S5E} mutant strains.

(A) The *cofilin*^{S5A} mutant is accomplished by GCA instead of TCC in native locus of *cofilin* gene and the pyrithiamine gene is selected as the resistance marker. **(B)** The *cofilin* sequences of WT, *cofilin*^{S5A} mutant cassette and *cofilin*^{S5A} strain were aligned by DNAMAN Version 7. The red boxes indicate the changed nucleotides in *cofilin* gene. **(C)** The map of plasmid pLH2 which is used to express cofilin^{S5E} is shown and the GFP protein is fused into N-terminal of cofilin. **(D)** The plasmid pLH2 were transformed into *cofilin*$_{teton}$ strain successfully to produce *cofilin*$_{teton}$/*cofilin*^{S5E} strain, which showed green fluorescence. Scale bar, 50 μm.

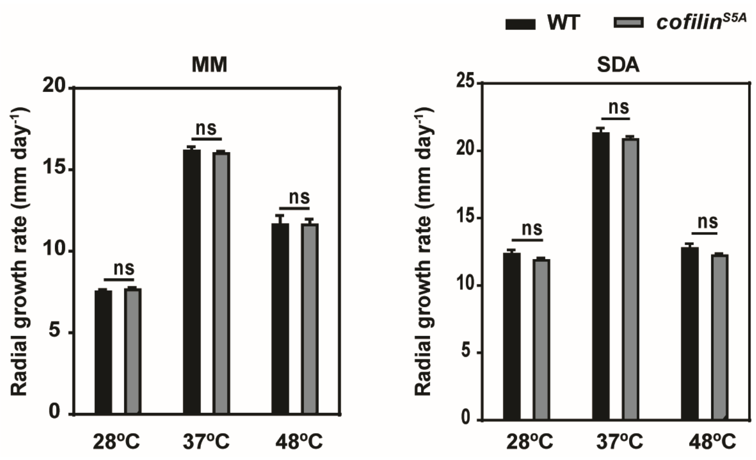

Figure S4. Growth of the *cofilin^{S5A}* strain.

3×10^5 conidia of *cofilin^{S5A}* and WT were spotted in the center of solid AMM medium and incubated at 28°C, 37°C and 48°C, respectively. The growth rates were determined as the increase in colony diameter per day (mm day^{-1}). Data are represented as mean ± SE (n = 3).

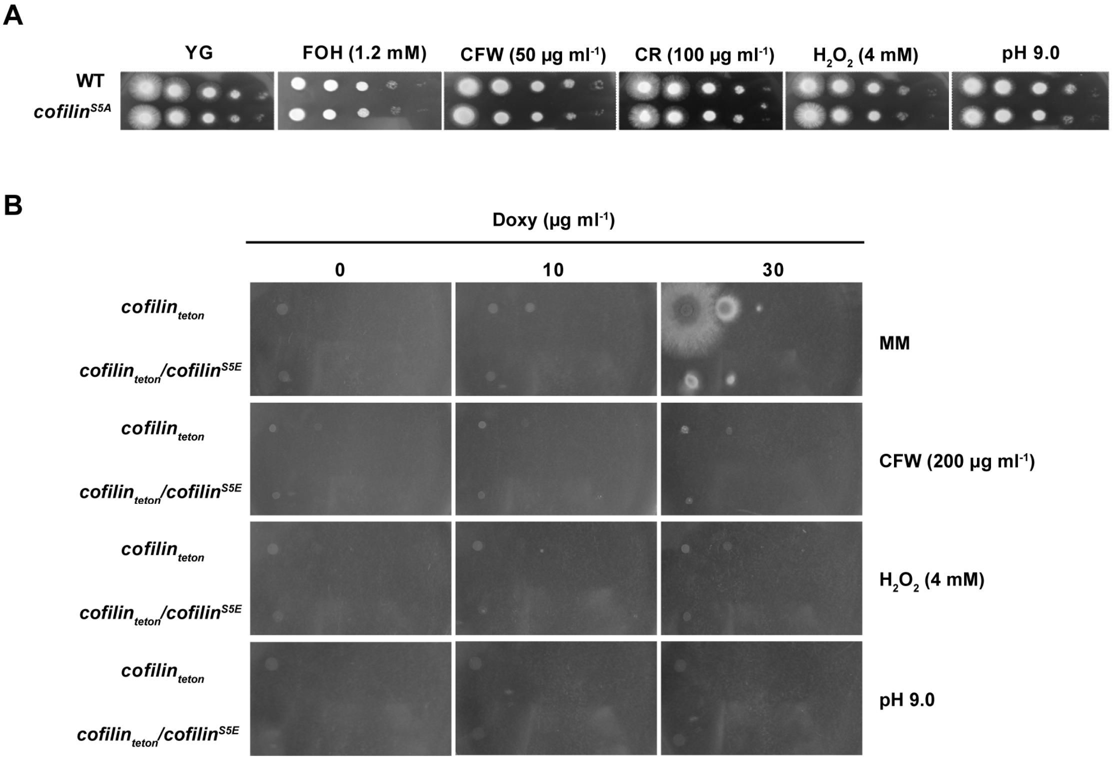

Figure S5. Growth of *cofilin^{S5A}* and *cofilin$_{teton}$/cofilin^{S5E}* mutant strains under different stresses.

(A) In a series of 10-fold dilutions derived from a starting suspension of 1×10^8 conidia ml^{-1} of the indicated strains, aliquots of 2 µl were spotted on YG with or without cell wall perturbing agents, H_2O_2 and at pH 9.0. **(B)** In a series of 10-fold dilutions derived from a starting suspension of 1×10^8 conidia ml^{-1} of the indicated strains, aliquots of 2

µl were spotted on AMM containing the amount of doxycycline with or without cell wall perturbing agents, H_2O_2 and at different pH. After a 48 h incubation at 37°C, the colony growth was comparatively analyzed. The results shown are representative of 3 experiments.
